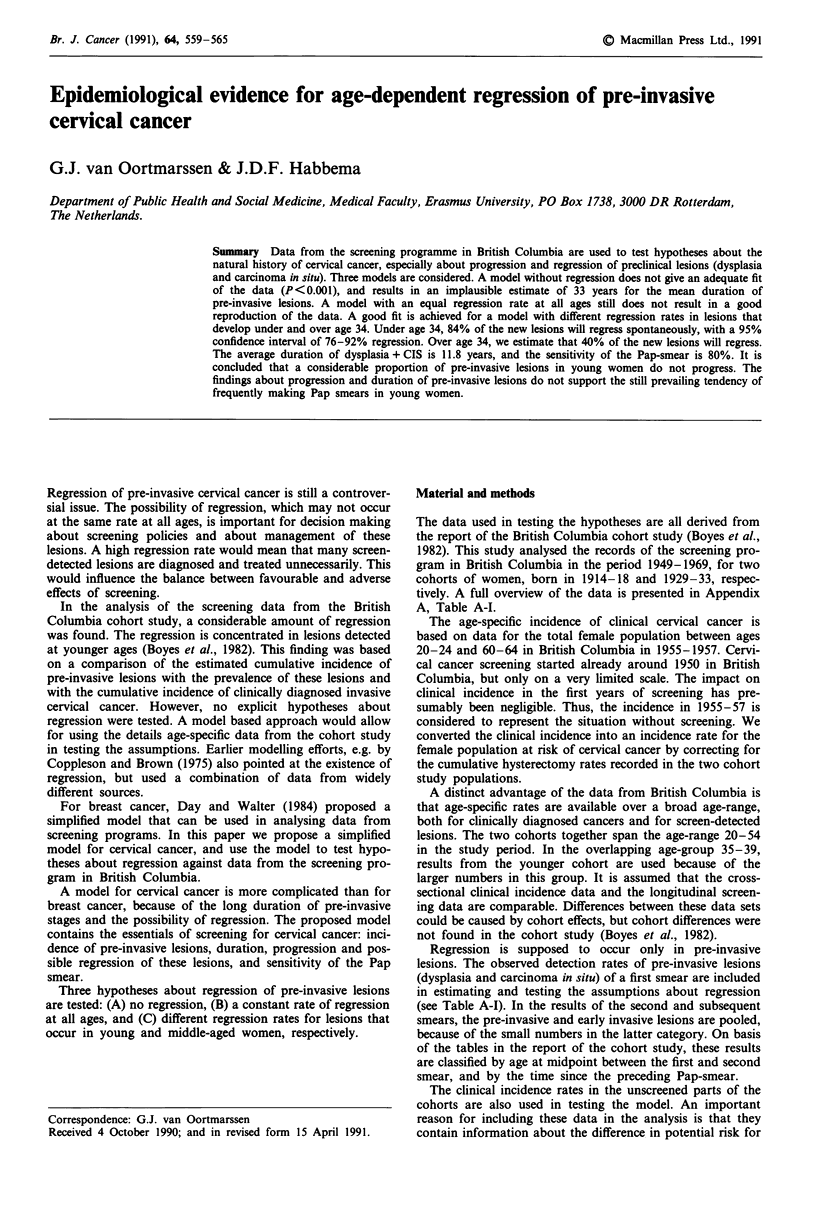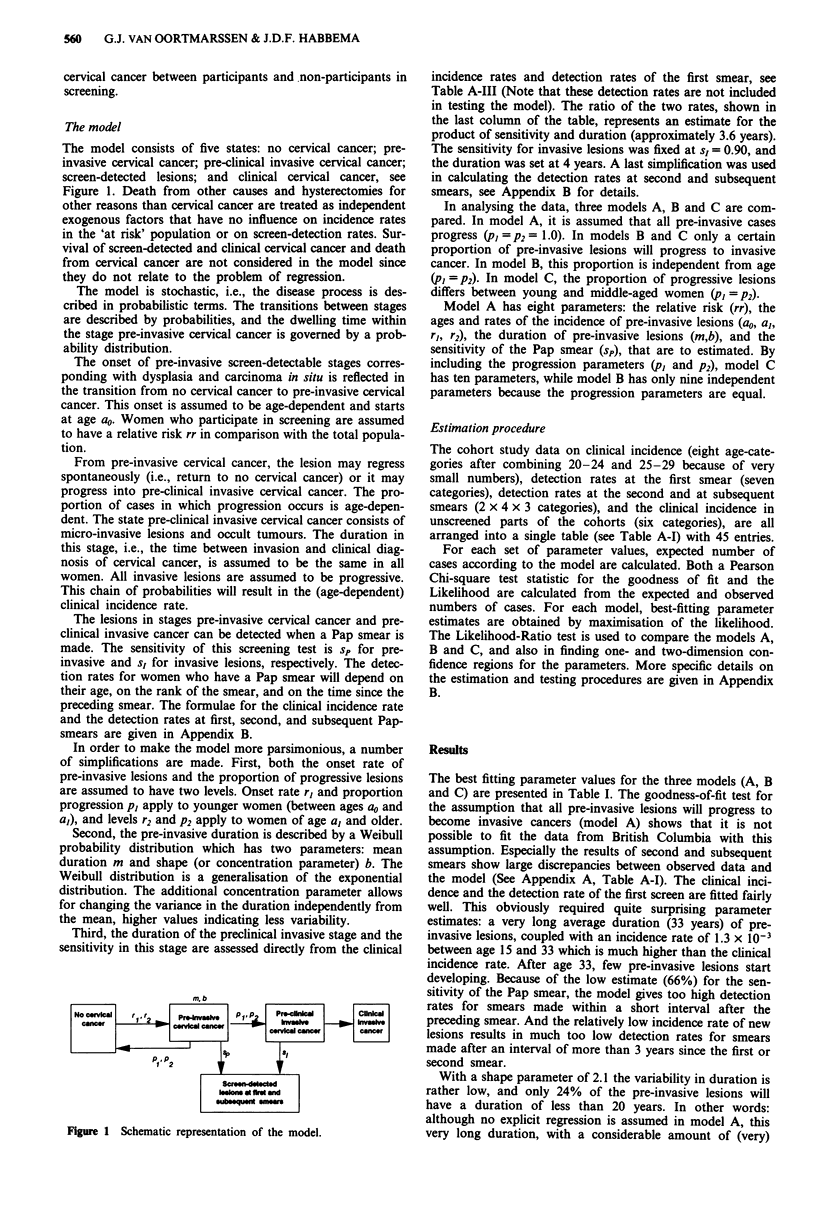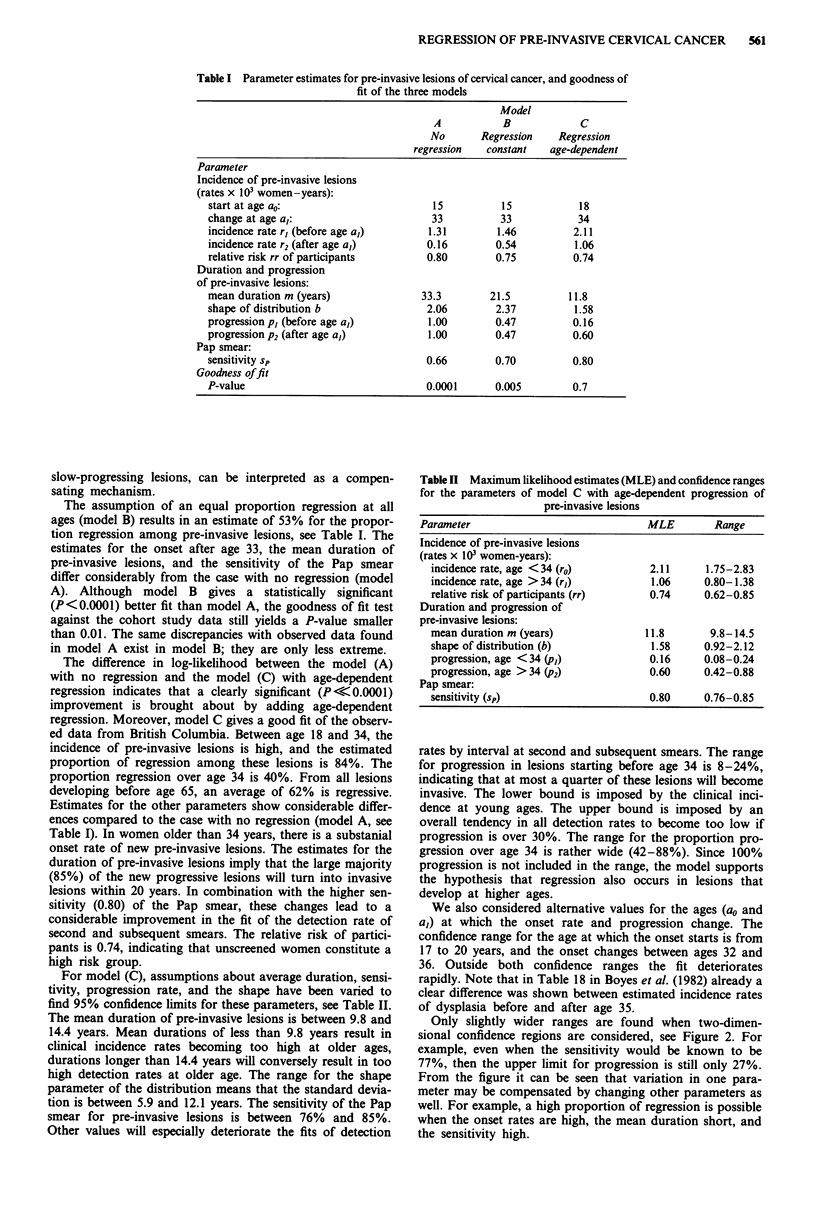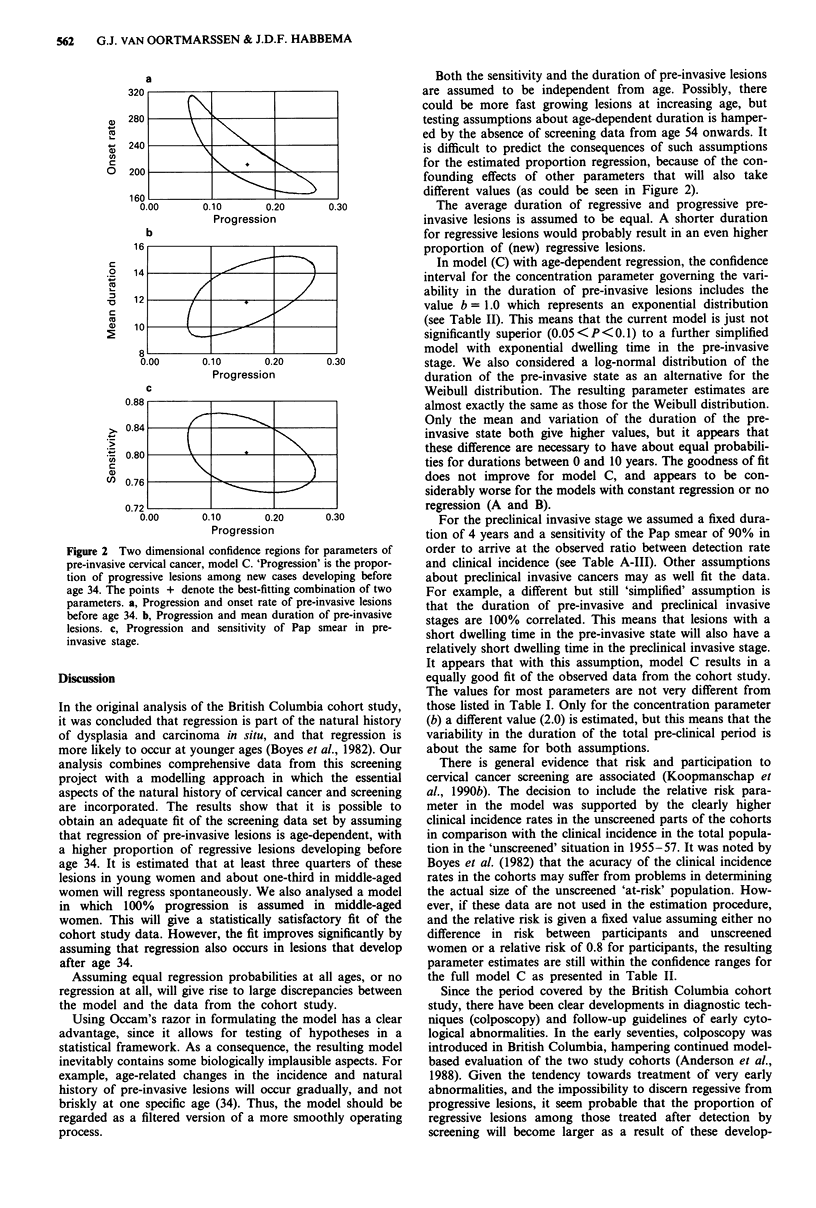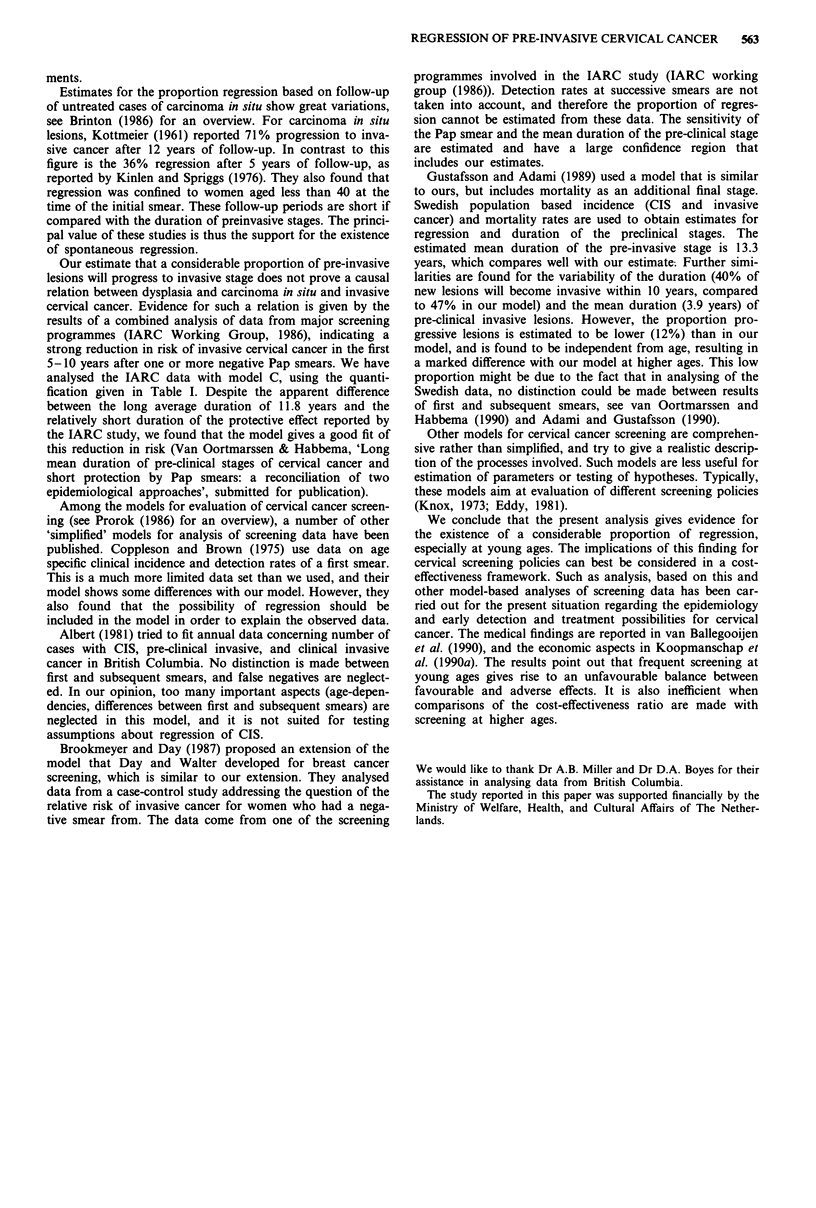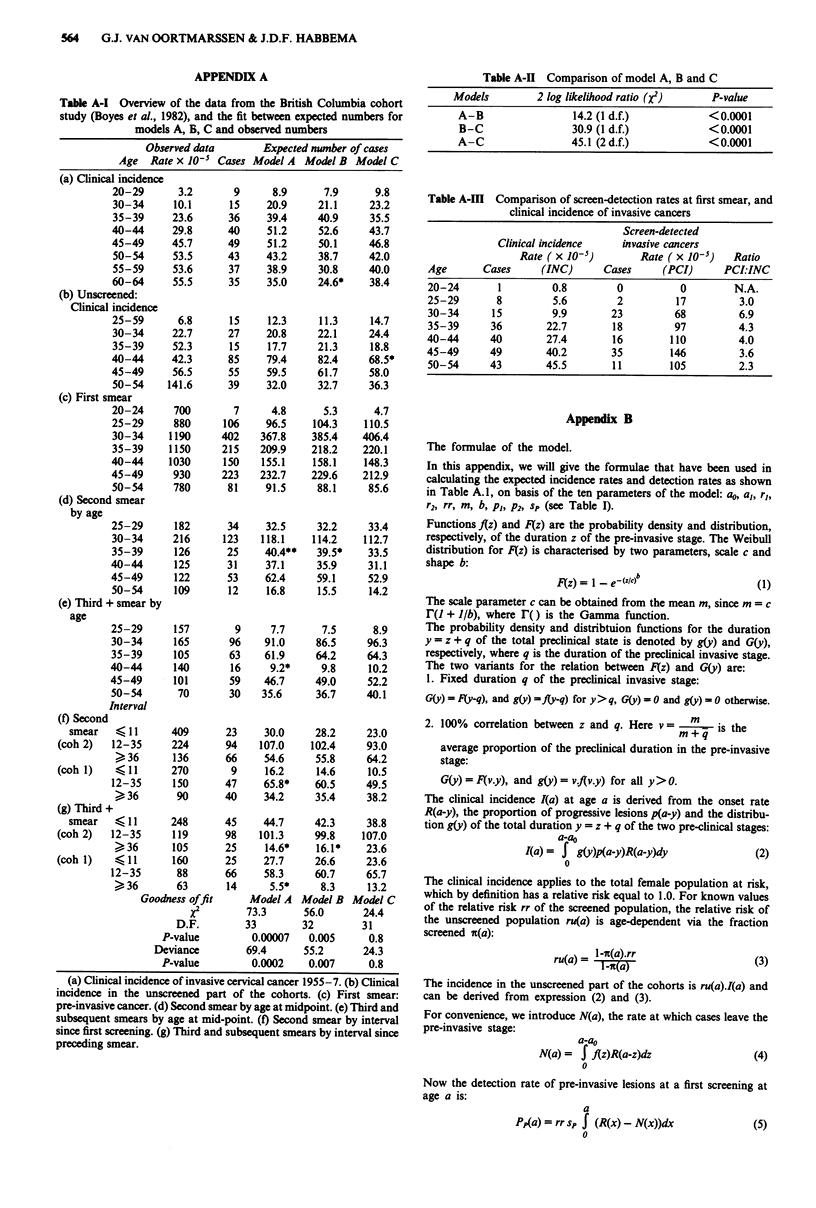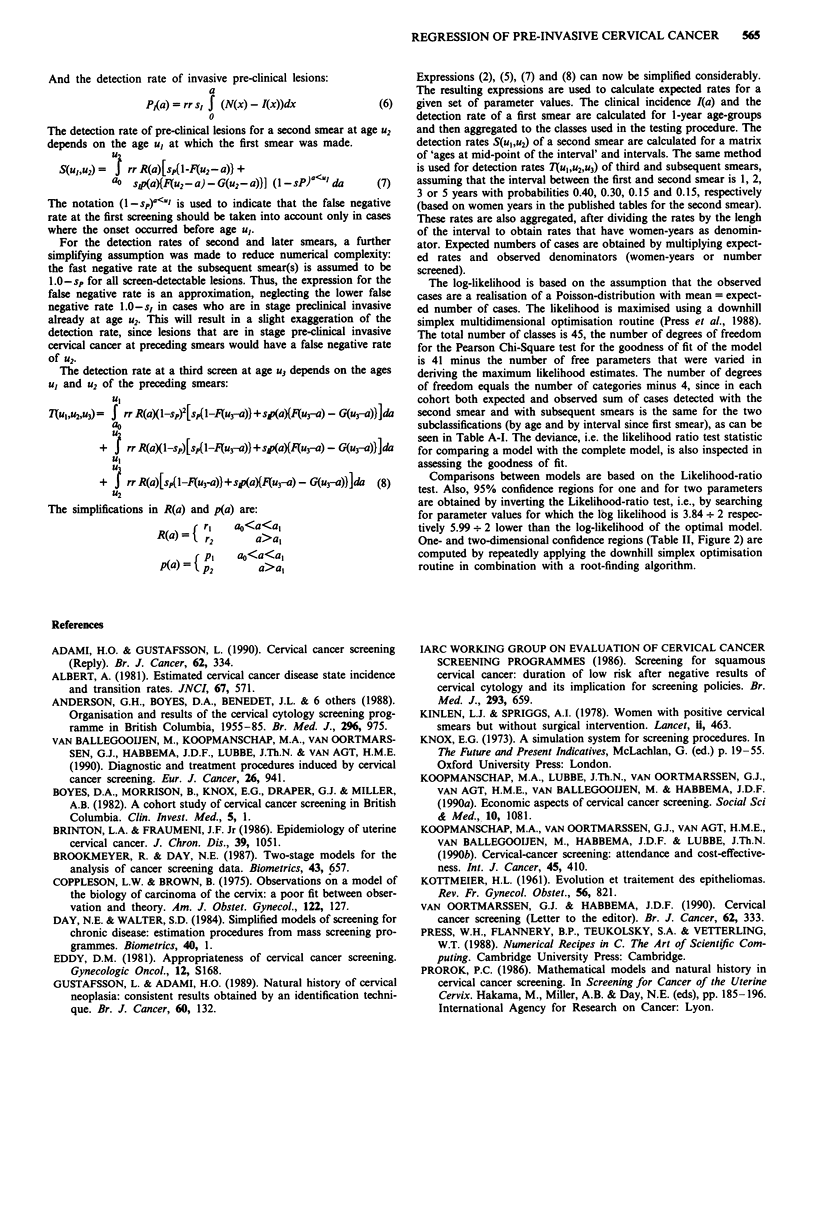# Epidemiological evidence for age-dependent regression of pre-invasive cervical cancer.

**DOI:** 10.1038/bjc.1991.350

**Published:** 1991-09

**Authors:** G. J. van Oortmarssen, J. D. Habbema

**Affiliations:** Department of Public Health and Social Medicine, Medical Faculty, Erasmus University, Rotterdam, The Netherlands.

## Abstract

Data from the screening programme in British Columbia are used to test hypotheses about the natural history of cervical cancer, especially about progression and regression of preclinical lesions (dysplasia and carcinoma in situ). Three models are considered. A model without regression does not give an adequate fit of the data (P less than 0.001), and results in an implausible estimate of 33 years for the mean duration of pre-invasive lesions. A model with an equal regression rate at all ages still does not result in a good reproduction of the data. A good fit is achieved for a model with different regression rates in lesions that develop under and over age 34. Under age 34, 84% of the new lesions will regress spontaneously, with a 95% confidence interval of 76-92% regression. Over age 34, we estimate that 40% of the new lesions will regress. The average duration of dysplasia + CIS is 11.8 years, and the sensitivity of the Pap-smear is 80%. It is concluded that a considerable proportion of pre-invasive lesions in young women do not progress. The findings about progression and duration of pre-invasive lesions do not support the still prevailing tendency of frequently making Pap smears in young women.


Br. J. Cancer (1991), 64, 559–565 © Macmillan Press Ltd., 1991

### Epidemiological evidence for age-dependent regression of pre-invasive cervical cancer

G.J. van Oortmarssen & J.D.F. Habbema

*Department of Public Health and Social Medicine, Medical Faculty, Erasmus University, PO Box 1738, 3000 DR Rotterdam, The Netherlands.*

**Summary** Data from the screening programme in British Columbia are used to test hypotheses about the natural history of cervical cancer, especially about progression and regression of preclinical lesions (dysplasia and carcinoma *in situ*). Three models are considered. A model without regression does not give an adequate fit of the data ($P < 0.001$), and results in an implausible estimate of 33 years for the mean duration of pre-invasive lesions. A model with an equal regression rate at all ages still does not result in a good reproduction of the data. A good fit is achieved for a model with different regression rates in lesions that develop under and over age 34. Under age 34, 84% of the new lesions will regress spontaneously, with a 95% confidence interval of 76–92% regression. Over age 34, we estimate that 40% of the new lesions will regress. The average duration of dysplasia + CIS is 11.8 years, and the sensitivity of the Pap-smear is 80%. It is concluded that a considerable proportion of pre-invasive lesions in young women do not progress. The findings about progression and duration of pre-invasive lesions do not support the still prevailing tendency of frequently making Pap smears in young women.

Regression of pre-invasive cervical cancer is still a controversial issue. The possibility of regression, which may not occur at the same rate at all ages, is important for decision making about screening policies and about management of these lesions. A high regression rate would mean that many screen-detected lesions are diagnosed and treated unnecessarily. This would influence the balance between favourable and adverse effects of screening.

In the analysis of the screening data from the British Columbia cohort study, a considerable amount of regression was found. The regression is concentrated in lesions detected at younger ages (Boyes *et al*., 1982). This finding was based on a comparison of the estimated cumulative incidence of pre-invasive lesions with the prevalence of these lesions and with the cumulative incidence of clinically diagnosed invasive cervical cancer. However, no explicit hypotheses about regression were tested. A model based approach would allow for using the details age-specific data from the cohort study in testing the assumptions. Earlier modelling efforts, e.g. by Coppleson and Brown (1975) also pointed at the existence of regression, but used a combination of data from widely different sources.

For breast cancer, Day and Walter (1984) proposed a simplified model that can be used in analysing data from screening programs. In this paper we propose a simplified model for cervical cancer, and use the model to test hypotheses about regression against data from the screening program in British Columbia.

A model for cervical cancer is more complicated than for breast cancer, because of the long duration of pre-invasive stages and the possibility of regression. The proposed model contains the essentials of screening for cervical cancer: incidence of pre-invasive lesions, duration, progression and possible regression of these lesions, and sensitivity of the Pap smear.

Three hypotheses about regression of pre-invasive lesions are tested: (A) no regression, (B) a constant rate of regression at all ages, and (C) different regression rates for lesions that occur in young and middle-aged women, respectively.

Correspondence: G.J. van Oortmarssen
Received 4 October 1990; and in revised form 15 April 1991.

### Material and methods

The data used in testing the hypotheses are all derived from the report of the British Columbia cohort study (Boyes *et al*., 1982). This study analysed the records of the screening program in British Columbia in the period 1949–1969, for two cohorts of women, born in 1914–18 and 1929–33, respectively. A full overview of the data is presented in Appendix A, Table A-I.

The age-specific incidence of clinical cervical cancer is based on data for the total female population between ages 20–24 and 60–64 in British Columbia in 1955–1957. Cervical cancer screening started already around 1950 in British Columbia, but only on a very limited scale. The impact on clinical incidence in the first years of screening has presumably been negligible. Thus, the incidence in 1955–57 is considered to represent the situation without screening. We converted the clinical incidence into an incidence rate for the female population at risk of cervical cancer by correcting for the cumulative hysterectomy rates recorded in the two cohort study populations.

A distinct advantage of the data from British Columbia is that age-specific rates are available over a broad age-range, both for clinically diagnosed cancers and for screen-detected lesions. The two cohorts together span the age-range 20–54 in the study period. In the overlapping age-group 35–39, results from the younger cohort are used because of the larger numbers in this group. It is assumed that the cross-sectional clinical incidence data and the longitudinal screening data are comparable. Differences between these data sets could be caused by cohort effects, but cohort differences were not found in the cohort study (Boyes *et al*., 1982).

Regression is supposed to occur only in pre-invasive lesions. The observed detection rates of pre-invasive lesions (dysplasia and carcinoma *in situ*) of a first smear are included in estimating and testing the assumptions about regression (see Table A-I). In the results of the second and subsequent smears, the pre-invasive and early invasive lesions are pooled, because of the small numbers in the latter category. On basis of the tables in the report of the cohort study, these results are classified by age at midpoint between the first and second smear, and by the time since the preceding Pap-smear.

The clinical incidence rates in the unscreened parts of the cohorts are also used in testing the model. An important reason for including these data in the analysis is that they contain information about the difference in potential risk for cervical cancer between participants and non-participants in screening.

### *The model*

The model consists of five states: no cervical cancer; pre-invasive cervical cancer; pre-clinical invasive cervical cancer; screen-detected lesions; and clinical cervical cancer, see Figure 1. Death from other causes and hysterectomies for other reasons than cervical cancer are treated as independent exogenous factors that have no influence on incidence rates in the 'at risk' population or on screen-detection rates. Survival of screen-detected and clinical cervical cancer and death from cervical cancer are not considered in the model since they do not relate to the problem of regression.

The model is stochastic, i.e., the disease process is described in probabilistic terms. The transitions between stages are described by probabilities, and the dwelling time within the stage pre-invasive cervical cancer is governed by a probability distribution.

The onset of pre-invasive screen-detectable stages corresponding with dysplasia and carcinoma *in situ* is reflected in the transition from no cervical cancer to pre-invasive cervical cancer. This onset is assumed to be age-dependent and starts at age $a_0$. Women who participate in screening are assumed to have a relative risk $rr$ in comparison with the total population.

From pre-invasive cervical cancer, the lesion may regress spontaneously (i.e., return to no cervical cancer) or it may progress into pre-clinical invasive cervical cancer. The proportion of cases in which progression occurs is age-dependent. The state pre-clinical invasive cervical cancer consists of micro-invasive lesions and occult tumours. The duration in this stage, i.e., the time between invasion and clinical diagnosis of cervical cancer, is assumed to be the same in all women. All invasive lesions are assumed to be progressive. This chain of probabilities will result in the (age-dependent) clinical incidence rate.

The lesions in stages pre-invasive cervical cancer and pre-clinical invasive cancer can be detected when a Pap smear is made. The sensitivity of this screening test is $s_P$ for pre-invasive and $s_I$ for invasive lesions, respectively. The detection rates for women who have a Pap smear will depend on their age, on the rank of the smear, and on the time since the preceding smear. The formulae for the clinical incidence rate and the detection rates at first, second, and subsequent Pap-smears are given in Appendix B.

In order to make the model more parsimonious, a number of simplifications are made. First, both the onset rate of pre-invasive lesions and the proportion of progressive lesions are assumed to have two levels. Onset rate $r_1$ and proportion progression $p_1$ apply to younger women (between ages $a_0$ and $a_1$), and levels $r_2$ and $p_2$ apply to women of age $a_1$ and older.

Second, the pre-invasive duration is described by a Weibull probability distribution which has two parameters: mean duration $m$ and shape (or concentration parameter) $b$. The Weibull distribution is a generalisation of the exponential distribution. The additional concentration parameter allows for changing the variance in the duration independently from the mean, higher values indicating less variability.

Third, the duration of the preclinical invasive stage and the sensitivity in this stage are assessed directly from the clinical

**Figure 1** Schematic representation of the model.

incidence rates and detection rates of the first smear, see Table A-III (Note that these detection rates are not included in testing the model). The ratio of the two rates, shown in the last column of the table, represents an estimate for the product of sensitivity and duration (approximately 3.6 years). The sensitivity for invasive lesions was fixed at $s_I = 0.90$, and the duration was set at 4 years. A last simplification was used in calculating the detection rates at second and subsequent smears, see Appendix B for details.

In analysing the data, three models A, B and C are compared. In model A, it is assumed that all pre-invasive cases progress ($p_1 = p_2 = 1.0$). In models B and C only a certain proportion of pre-invasive lesions will progress to invasive cancer. In model B, this proportion is independent from age ($p_1 = p_2$). In model C, the proportion of progressive lesions differs between young and middle-aged women ($p_1 = p_2$).

Model A has eight parameters: the relative risk ($rr$), the ages and rates of the incidence of pre-invasive lesions ($a_0$, $a_1$, $r_1$, $r_2$), the duration of pre-invasive lesions ($m$,$b$), and the sensitivity of the Pap smear ($s_P$), that are to estimated. By including the progression parameters ($p_1$ and $p_2$), model C has ten parameters, while model B has only nine independent parameters because the progression parameters are equal.

### *Estimation procedure*

The cohort study data on clinical incidence (eight age-categories after combining 20–24 and 25–29 because of very small numbers), detection rates at the first smear (seven categories), detection rates at the second and at subsequent smears ($2 \times 4 \times 3$ categories), and the clinical incidence in unscreened parts of the cohorts (six categories), are all arranged into a single table (see Table A-I) with 45 entries.

For each set of parameter values, expected number of cases according to the model are calculated. Both a Pearson Chi-square test statistic for the goodness of fit and the Likelihood are calculated from the expected and observed numbers of cases. For each model, best-fitting parameter estimates are obtained by maximisation of the likelihood. The Likelihood-Ratio test is used to compare the models A, B and C, and also in finding one- and two-dimension confidence regions for the parameters. More specific details on the estimation and testing procedures are given in Appendix B.

### Results

The best fitting parameter values for the three models (A, B and C) are presented in Table I. The goodness-of-fit test for the assumption that all pre-invasive lesions will progress to become invasive cancers (model A) shows that it is not possible to fit the data from British Columbia with this assumption. Especially the results of second and subsequent smears show large discrepancies between observed data and the model (See Appendix A, Table A-I). The clinical incidence and the detection rate of the first screen are fitted fairly well. This obviously required quite surprising parameter estimates: a very long average duration (33 years) of pre-invasive lesions, coupled with an incidence rate of $1.3 \times 10^{-3}$ between age 15 and 33 which is much higher than the clinical incidence rate. After age 33, few pre-invasive lesions start developing. Because of the low estimate (66%) for the sensitivity of the Pap smear, the model gives too high detection rates for smears made within a short interval after the preceding smear. And the relatively low incidence rate of new lesions results in much too low detection rates for smears made after an interval of more than 3 years since the first or second smear.

With a shape parameter of 2.1 the variability in duration is rather low, and only 24% of the pre-invasive lesions will have a duration of less than 20 years. In other words: although no explicit regression is assumed in model A, this very long duration, with a considerable amount of (very)

**Table I** Parameter estimates for pre-invasive lesions of cervical cancer, and goodness of fit of the three models

| | *Model* A No regression | *Model* B Regression constant | *Model* C Regression age-dependent |
|---|---|---|---|
| *Parameter* | | | |
| Incidence of pre-invasive lesions (rates × $10^3$ women–years): | | | |
| start at age $a_0$: | 15 | 15 | 18 |
| change at age $a_l$: | 33 | 33 | 34 |
| incidence rate $r_l$ (before age $a_l$) | 1.31 | 1.46 | 2.11 |
| incidence rate $r_2$ (after age $a_l$) | 0.16 | 0.54 | 1.06 |
| relative risk $rr$ of participants | 0.80 | 0.75 | 0.74 |
| Duration and progression of pre-invasive lesions: | | | |
| mean duration $m$ (years) | 33.3 | 21.5 | 11.8 |
| shape of distribution $b$ | 2.06 | 2.37 | 1.58 |
| progression $p_l$ (before age $a_l$) | 1.00 | 0.47 | 0.16 |
| progression $p_2$ (after age $a_l$) | 1.00 | 0.47 | 0.60 |
| Pap smear: | | | |
| sensitivity $s_P$ | 0.66 | 0.70 | 0.80 |
| *Goodness of fit* | | | |
| *P*-value | 0.0001 | 0.005 | 0.7 |

slow-progressing lesions, can be interpreted as a compensating mechanism.

The assumption of an equal proportion regression at all ages (model B) results in an estimate of 53% for the proportion regression among pre-invasive lesions, see Table I. The estimates for the onset after age 33, the mean duration of pre-invasive lesions, and the sensitivity of the Pap smear differ considerably from the case with no regression (model A). Although model B gives a statistically significant ($P<0.0001$) better fit than model A, the goodness of fit test against the cohort study data still yields a *P*-value smaller than 0.01. The same discrepancies with observed data found in model A exist in model B; they are only less extreme.

The difference in log-likelihood between the model (A) with no regression and the model (C) with age-dependent regression indicates that a clearly significant ($P\ll0.0001$) improvement is brought about by adding age-dependent regression. Moreover, model C gives a good fit of the observed data from British Columbia. Between age 18 and 34, the incidence of pre-invasive lesions is high, and the estimated proportion of regression among these lesions is 84%. The proportion regression over age 34 is 40%. From all lesions developing before age 65, an average of 62% is regressive. Estimates for the other parameters show considerable differences compared to the case with no regression (model A, see Table I). In women older than 34 years, there is a substanial onset rate of new pre-invasive lesions. The estimates for the duration of pre-invasive lesions imply that the large majority (85%) of the new progressive lesions will turn into invasive lesions within 20 years. In combination with the higher sensitivity (0.80) of the Pap smear, these changes lead to a considerable improvement in the fit of the detection rate of second and subsequent smears. The relative risk of participants is 0.74, indicating that unscreened women constitute a high risk group.

For model (C), assumptions about average duration, sensitivity, progression rate, and the shape have been varied to find 95% confidence limits for these parameters, see Table II. The mean duration of pre-invasive lesions is between 9.8 and 14.4 years. Mean durations of less than 9.8 years result in clinical incidence rates becoming too high at older ages, durations longer than 14.4 years will conversely result in too high detection rates at older age. The range for the shape parameter of the distribution means that the standard deviation is between 5.9 and 12.1 years. The sensitivity of the Pap smear for pre-invasive lesions is between 76% and 85%. Other values will especially deteriorate the fits of detection

**Table II** Maximum likelihood estimates (MLE) and confidence ranges for the parameters of model C with age-dependent progression of pre-invasive lesions

| *Parameter* | *MLE* | *Range* |
|---|---|---|
| Incidence of pre-invasive lesions (rates × $10^3$ women-years): | | |
| incidence rate, age $<34$ ($r_0$) | 2.11 | 1.75–2.83 |
| incidence rate, age $>34$ ($r_l$) | 1.06 | 0.80–1.38 |
| relative risk of participants ($rr$) | 0.74 | 0.62–0.85 |
| Duration and progression of pre-invasive lesions: | | |
| mean duration $m$ (years) | 11.8 | 9.8–14.5 |
| shape of distribution ($b$) | 1.58 | 0.92–2.12 |
| progression, age $<34$ ($p_l$) | 0.16 | 0.08–0.24 |
| progression, age $>34$ ($p_2$) | 0.60 | 0.42–0.88 |
| Pap smear: | | |
| sensitivity ($s_P$) | 0.80 | 0.76–0.85 |

rates by interval at second and subsequent smears. The range for progression in lesions starting before age 34 is 8–24%, indicating that at most a quarter of these lesions will become invasive. The lower bound is imposed by the clinical incidence at young ages. The upper bound is imposed by an overall tendency in all detection rates to become too low if progression is over 30%. The range for the proportion progression over age 34 is rather wide (42–88%). Since 100% progression is not included in the range, the model supports the hypothesis that regression also occurs in lesions that develop at higher ages.

We also considered alternative values for the ages ($a_0$ and $a_l$) at which the onset rate and progression change. The confidence range for the age at which the onset starts is from 17 to 20 years, and the onset changes between ages 32 and 36. Outside both confidence ranges the fit deteriorates rapidly. Note that in Table 18 in Boyes *et al.* (1982) already a clear difference was shown between estimated incidence rates of dysplasia before and after age 35.

Only slightly wider ranges are found when two-dimensional confidence regions are considered, see Figure 2. For example, even when the sensitivity would be known to be 77%, then the upper limit for progression is still only 27%. From the figure it can be seen that variation in one parameter may be compensated by changing other parameters as well. For example, a high proportion of regression is possible when the onset rates are high, the mean duration short, and the sensitivity high.

**Figure 2** Two dimensional confidence regions for parameters of pre-invasive cervical cancer, model C. 'Progression' is the proportion of progressive lesions among new cases developing before age 34. The points + denote the best-fitting combination of two parameters. **a**, Progression and onset rate of pre-invasive lesions before age 34. **b**, Progression and mean duration of pre-invasive lesions. **c**, Progression and sensitivity of Pap smear in pre-invasive stage.

### Discussion

In the original analysis of the British Columbia cohort study, it was concluded that regression is part of the natural history of dysplasia and carcinoma *in situ*, and that regression is more likely to occur at younger ages (Boyes *et al.*, 1982). Our analysis combines comprehensive data from this screening project with a modelling approach in which the essential aspects of the natural history of cervical cancer and screening are incorporated. The results show that it is possible to obtain an adequate fit of the screening data set by assuming that regression of pre-invasive lesions is age-dependent, with a higher proportion of regressive lesions developing before age 34. It is estimated that at least three quarters of these lesions in young women and about one-third in middle-aged women will regress spontaneously. We also analysed a model in which 100% progression is assumed in middle-aged women. This will give a statistically satisfactory fit of the cohort study data. However, the fit improves significantly by assuming that regression also occurs in lesions that develop after age 34.

Assuming equal regression probabilities at all ages, or no regression at all, will give rise to large discrepancies between the model and the data from the cohort study.

Using Occam's razor in formulating the model has a clear advantage, since it allows for testing of hypotheses in a statistical framework. As a consequence, the resulting model inevitably contains some biologically implausible aspects. For example, age-related changes in the incidence and natural history of pre-invasive lesions will occur gradually, and not briskly at one specific age (34). Thus, the model should be regarded as a filtered version of a more smoothly operating process.

Both the sensitivity and the duration of pre-invasive lesions are assumed to be independent from age. Possibly, there could be more fast growing lesions at increasing age, but testing assumptions about age-dependent duration is hampered by the absence of screening data from age 54 onwards. It is difficult to predict the consequences of such assumptions for the estimated proportion regression, because of the confounding effects of other parameters that will also take different values (as could be seen in Figure 2).

The average duration of regressive and progressive pre-invasive lesions is assumed to be equal. A shorter duration for regressive lesions would probably result in an even higher proportion of (new) regressive lesions.

In model (C) with age-dependent regression, the confidence interval for the concentration parameter governing the variability in the duration of pre-invasive lesions includes the value $b = 1.0$ which represents an exponential distribution (see Table II). This means that the current model is just not significantly superior ($0.05 < P < 0.1$) to a further simplified model with exponential dwelling time in the pre-invasive stage. We also considered a log-normal distribution of the duration of the pre-invasive state as an alternative for the Weibull distribution. The resulting parameter estimates are almost exactly the same as those for the Weibull distribution. Only the mean and variation of the duration of the pre-invasive state both give higher values, but it appears that these difference are necessary to have about equal probabilities for durations between 0 and 10 years. The goodness of fit does not improve for model C, and appears to be considerably worse for the models with constant regression or no regression (A and B).

For the preclinical invasive stage we assumed a fixed duration of 4 years and a sensitivity of the Pap smear of 90% in order to arrive at the observed ratio between detection rate and clinical incidence (see Table A-III). Other assumptions about preclinical invasive cancers may as well fit the data. For example, a different but still 'simplified' assumption is that the duration of pre-invasive and preclinical invasive stages are 100% correlated. This means that lesions with a short dwelling time in the pre-invasive state will also have a relatively short dwelling time in the preclinical invasive stage. It appears that with this assumption, model C results in a equally good fit of the observed data from the cohort study. The values for most parameters are not very different from those listed in Table I. Only for the concentration parameter ($b$) a different value (2.0) is estimated, but this means that the variability in the duration of the total pre-clinical period is about the same for both assumptions.

There is general evidence that risk and participation to cervical cancer screening are associated (Koopmanschap *et al.*, 1990*b*). The decision to include the relative risk parameter in the model was supported by the clearly higher clinical incidence rates in the unscreened parts of the cohorts in comparison with the clinical incidence in the total population in the 'unscreened' situation in 1955–57. It was noted by Boyes *et al.* (1982) that the acuracy of the clinical incidence rates in the cohorts may suffer from problems in determining the actual size of the unscreened 'at-risk' population. However, if these data are not used in the estimation procedure, and the relative risk is given a fixed value assuming either no difference in risk between participants and unscreened women or a relative risk of 0.8 for participants, the resulting parameter estimates are still within the confidence ranges for the full model C as presented in Table II.

Since the period covered by the British Columbia cohort study, there have been clear developments in diagnostic techniques (colposcopy) and follow-up guidelines of early cytological abnormalities. In the early seventies, colposcopy was introduced in British Columbia, hampering continued model-based evaluation of the two study cohorts (Anderson *et al.*, 1988). Given the tendency towards treatment of very early abnormalities, and the impossibility to discern regessive from progressive lesions, it seem probable that the proportion of regressive lesions among those treated after detection by screening will become larger as a result of these developments.

Estimates for the proportion regression based on follow-up of untreated cases of carcinoma *in situ* show great variations, see Brinton (1986) for an overview. For carcinoma *in situ* lesions, Kottmeier (1961) reported 71% progression to invasive cancer after 12 years of follow-up. In contrast to this figure is the 36% regression after 5 years of follow-up, as reported by Kinlen and Spriggs (1976). They also found that regression was confined to women aged less than 40 at the time of the initial smear. These follow-up periods are short if compared with the duration of preinvasive stages. The principal value of these studies is thus the support for the existence of spontaneous regression.

Our estimate that a considerable proportion of pre-invasive lesions will progress to invasive stage does not prove a causal relation between dysplasia and carcinoma *in situ* and invasive cervical cancer. Evidence for such a relation is given by the results of a combined analysis of data from major screening programmes (IARC Working Group, 1986), indicating a strong reduction in risk of invasive cervical cancer in the first 5–10 years after one or more negative Pap smears. We have analysed the IARC data with model C, using the quantification given in Table I. Despite the apparent difference between the long average duration of 11.8 years and the relatively short duration of the protective effect reported by the IARC study, we found that the model gives a good fit of this reduction in risk (Van Oortmarssen & Habbema, 'Long mean duration of pre-clinical stages of cervical cancer and short protection by Pap smears: a reconciliation of two epidemiological approaches', submitted for publication).

Among the models for evaluation of cervical cancer screening (see Prorok (1986) for an overview), a number of other 'simplified' models for analysis of screening data have been published. Coppleson and Brown (1975) use data on age specific clinical incidence and detection rates of a first smear. This is a much more limited data set than we used, and their model shows some differences with our model. However, they also found that the possibility of regression should be included in the model in order to explain the observed data.

Albert (1981) tried to fit annual data concerning number of cases with CIS, pre-clinical invasive, and clinical invasive cancer in British Columbia. No distinction is made between first and subsequent smears, and false negatives are neglected. In our opinion, too many important aspects (age-dependencies, differences between first and subsequent smears) are neglected in this model, and it is not suited for testing assumptions about regression of CIS.

Brookmeyer and Day (1987) proposed an extension of the model that Day and Walter developed for breast cancer screening, which is similar to our extension. They analysed data from a case-control study addressing the question of the relative risk of invasive cancer for women who had a negative smear from. The data come from one of the screening programmes involved in the IARC study (IARC working group (1986)). Detection rates at successive smears are not taken into account, and therefore the proportion of regression cannot be estimated from these data. The sensitivity of the Pap smear and the mean duration of the pre-clinical stage are estimated and have a large confidence region that includes our estimates.

Gustafsson and Adami (1989) used a model that is similar to ours, but includes mortality as an additional final stage. Swedish population based incidence (CIS and invasive cancer) and mortality rates are used to obtain estimates for regression and duration of the preclinical stages. The estimated mean duration of the pre-invasive stage is 13.3 years, which compares well with our estimate. Further similarities are found for the variability of the duration (40% of new lesions will become invasive within 10 years, compared to 47% in our model) and the mean duration (3.9 years) of pre-clinical invasive lesions. However, the proportion progressive lesions is estimated to be lower (12%) than in our model, and is found to be independent from age, resulting in a marked difference with our model at higher ages. This low proportion might be due to the fact that in analysing of the Swedish data, no distinction could be made between results of first and subsequent smears, see van Oortmarssen and Habbema (1990) and Adami and Gustafsson (1990).

Other models for cervical cancer screening are comprehensive rather than simplified, and try to give a realistic description of the processes involved. Such models are less useful for estimation of parameters or testing of hypotheses. Typically, these models aim at evaluation of different screening policies (Knox, 1973; Eddy, 1981).

We conclude that the present analysis gives evidence for the existence of a considerable proportion of regression, especially at young ages. The implications of this finding for cervical screening policies can best be considered in a cost-effectiveness framework. Such as analysis, based on this and other model-based analyses of screening data has been carried out for the present situation regarding the epidemiology and early detection and treatment possibilities for cervical cancer. The medical findings are reported in van Ballegooijen *et al.* (1990), and the economic aspects in Koopmanschap *et al.* (1990*a*). The results point out that frequent screening at young ages gives rise to an unfavourable balance between favourable and adverse effects. It is also inefficient when comparisons of the cost-effectiveness ratio are made with screening at higher ages.

We would like to thank Dr A.B. Miller and Dr D.A. Boyes for their assistance in analysing data from British Columbia.

The study reported in this paper was supported financially by the Ministry of Welfare, Health, and Cultural Affairs of The Netherlands.

### APPENDIX A

**Table A-I** Overview of the data from the British Columbia cohort study (Boyes *et al.*, 1982), and the fit between expected numbers for models A, B, C and observed numbers

| | *Observed data* | | *Expected number of cases* | | |
|---|---|---|---|---|---|
| *Age* | *Rate* × $10^{-5}$ | *Cases* | *Model A* | *Model B* | *Model C* |
| (a) Clinical incidence | | | | | |
| 20–29 | 3.2 | 9 | 8.9 | 7.9 | 9.8 |
| 30–34 | 10.1 | 15 | 20.9 | 21.1 | 23.2 |
| 35–39 | 23.6 | 36 | 39.4 | 40.9 | 35.5 |
| 40–44 | 29.8 | 40 | 51.2 | 52.6 | 43.7 |
| 45–49 | 45.7 | 49 | 51.2 | 50.1 | 46.8 |
| 50–54 | 53.5 | 43 | 43.2 | 38.7 | 42.0 |
| 55–59 | 53.6 | 37 | 38.9 | 30.8 | 40.0 |
| 60–64 | 55.5 | 35 | 35.0 | 24.6* | 38.4 |
| (b) Unscreened: Clinical incidence | | | | | |
| 25–59 | 6.8 | 15 | 12.3 | 11.3 | 14.7 |
| 30–34 | 22.7 | 27 | 20.8 | 22.1 | 24.4 |
| 35–39 | 52.3 | 15 | 17.7 | 21.3 | 18.8 |
| 40–44 | 42.3 | 85 | 79.4 | 82.4 | 68.5* |
| 45–49 | 56.5 | 55 | 59.5 | 61.7 | 58.0 |
| 50–54 | 141.6 | 39 | 32.0 | 32.7 | 36.3 |
| (c) First smear | | | | | |
| 20–24 | 700 | 7 | 4.8 | 5.3 | 4.7 |
| 25–29 | 880 | 106 | 96.5 | 104.3 | 110.5 |
| 30–34 | 1190 | 402 | 367.8 | 385.4 | 406.4 |
| 35–39 | 1150 | 215 | 209.9 | 218.2 | 220.1 |
| 40–44 | 1030 | 150 | 155.1 | 158.1 | 148.3 |
| 45–49 | 930 | 223 | 232.7 | 229.6 | 212.9 |
| 50–54 | 780 | 81 | 91.5 | 88.1 | 85.6 |
| (d) Second smear by age | | | | | |
| 25–29 | 182 | 34 | 32.5 | 32.2 | 33.4 |
| 30–34 | 216 | 123 | 118.1 | 114.2 | 112.7 |
| 35–39 | 126 | 25 | 40.4** | 39.5* | 33.5 |
| 40–44 | 125 | 31 | 37.1 | 35.9 | 31.1 |
| 45–49 | 122 | 53 | 62.4 | 59.1 | 52.9 |
| 50–54 | 109 | 12 | 16.8 | 15.5 | 14.2 |
| (e) Third + smear by age | | | | | |
| 25–29 | 157 | 9 | 7.7 | 7.5 | 8.9 |
| 30–34 | 165 | 96 | 91.0 | 86.5 | 96.3 |
| 35–39 | 105 | 63 | 61.9 | 64.2 | 64.3 |
| 40–44 | 140 | 16 | 9.2* | 9.8 | 10.2 |
| 45–49 | 101 | 59 | 46.7 | 49.0 | 52.2 |
| 50–54 | 70 | 30 | 35.6 | 36.7 | 40.1 |
| *Interval* | | | | | |
| (f) Second smear (coh 2) ⩽11 | 409 | 23 | 30.0 | 28.2 | 23.0 |
| (coh 2) 12–35 | 224 | 94 | 107.0 | 102.4 | 93.0 |
| ⩾36 | 136 | 66 | 54.6 | 55.8 | 64.2 |
| (coh 1) ⩽11 | 270 | 9 | 16.2 | 14.6 | 10.5 |
| 12–35 | 150 | 47 | 65.8* | 60.5 | 49.5 |
| ⩾36 | 90 | 40 | 34.2 | 35.4 | 38.2 |
| (g) Third + smear ⩽11 | 248 | 45 | 44.7 | 42.3 | 38.8 |
| (coh 2) 12–35 | 119 | 98 | 101.3 | 99.8 | 107.0 |
| ⩾36 | 105 | 25 | 14.6* | 16.1* | 23.6 |
| (coh 1) ⩽11 | 160 | 25 | 27.7 | 26.6 | 23.6 |
| 12–35 | 88 | 66 | 58.3 | 60.7 | 65.7 |
| ⩾36 | 63 | 14 | 5.5* | 8.3 | 13.2 |
| | *Goodness of fit* | | *Model A* | *Model B* | *Model C* |
| | $\chi^2$ | | 73.3 | 56.0 | 24.4 |
| | D.F. | | 33 | 32 | 31 |
| | *P*-value | | 0.00007 | 0.005 | 0.8 |
| | Deviance | | 69.4 | 55.2 | 24.3 |
| | *P*-value | | 0.0002 | 0.007 | 0.8 |

(a) Clinical incidence of invasive cervical cancer 1955–7. (b) Clinical incidence in the unscreened part of the cohorts. (c) First smear: pre-invasive cancer. (d) Second smear by age at midpoint. (e) Third and subsequent smears by age at mid-point. (f) Second smear by interval since first screening. (g) Third and subsequent smears by interval since preceding smear.

**Table A-II** Comparison of model A, B and C

| *Models* | *2 log likelihood ratio* ($\chi^2$) | *P-value* |
|---|---|---|
| A–B | 14.2 (1 d.f.) | <0.0001 |
| B–C | 30.9 (1 d.f.) | <0.0001 |
| A–C | 45.1 (2 d.f.) | <0.0001 |

**Table A-III** Comparison of screen-detection rates at first smear, and clinical incidence of invasive cancers

| | *Clinical incidence* | | *Screen-detected invasive cancers* | | |
|---|---|---|---|---|---|
| *Age* | *Cases* | *Rate* ( × $10^{-5}$) *(INC)* | *Cases* | *Rate* ( × $10^{-5}$) *(PCI)* | *Ratio PCI:INC* |
| 20–24 | 1 | 0.8 | 0 | 0 | N.A. |
| 25–29 | 8 | 5.6 | 2 | 17 | 3.0 |
| 30–34 | 15 | 9.9 | 23 | 68 | 6.9 |
| 35–39 | 36 | 22.7 | 18 | 97 | 4.3 |
| 40–44 | 40 | 27.4 | 16 | 110 | 4.0 |
| 45–49 | 49 | 40.2 | 35 | 146 | 3.6 |
| 50–54 | 43 | 45.5 | 11 | 105 | 2.3 |

### Appendix B

The formulae of the model.

In this appendix, we will give the formulae that have been used in calculating the expected incidence rates and detection rates as shown in Table A.1, on basis of the ten parameters of the model: $a_0$, $a_1$, $r_1$, $r_2$, $rr$, $m$, $b$, $p_1$, $p_2$, $s_P$ (see Table I).

Functions $f(z)$ and $F(z)$ are the probability density and distribution, respectively, of the duration $z$ of the pre-invasive stage. The Weibull distribution for $F(z)$ is characterised by two parameters, scale $c$ and shape $b$:

$$F(z) = 1 - e^{-(z/c)^b} \quad (1)$$

The scale parameter $c$ can be obtained from the mean $m$, since $m = c\,\Gamma(1 + 1/b)$, where $\Gamma()$ is the Gamma function.

The probability density and distribtuion functions for the duration $y = z + q$ of the total preclinical state is denoted by $g(y)$ and $G(y)$, respectively, where $q$ is the duration of the preclinical invasive stage. The two variants for the relation between $F(z)$ and $G(y)$ are:

1. Fixed duration $q$ of the preclinical invasive stage:

$G(y) = F(y\text{-}q)$, and $g(y) = f(y\text{-}q)$ for $y > q$, $G(y) = 0$ and $g(y) = 0$ otherwise.

2. 100% correlation between $z$ and $q$. Here $v = \frac{m}{m+q}$ is the average proportion of the preclinical duration in the pre-invasive stage:

$G(y) = F(v.y)$, and $g(y) = v.f(v.y)$ for all $y > 0$.

The clinical incidence $I(a)$ at age $a$ is derived from the onset rate $R(a\text{-}y)$, the proportion of progressive lesions $p(a\text{-}y)$ and the distribution $g(y)$ of the total duration $y = z + q$ of the two pre-clinical stages:

$$I(a) = \int_0^{a-a_0} g(y)p(a\text{-}y)R(a\text{-}y)dy \quad (2)$$

The clinical incidence applies to the total female population at risk, which by definition has a relative risk equal to 1.0. For known values of the relative risk $rr$ of the screened population, the relative risk of the unscreened population $ru(a)$ is age-dependent via the fraction screened $\pi(a)$:

$$ru(a) = \frac{1\text{-}\pi(a).rr}{1\text{-}\pi(a)} \quad (3)$$

The incidence in the unscreened part of the cohorts is $ru(a).I(a)$ and can be derived from expression (2) and (3).

For convenience, we introduce $N(a)$, the rate at which cases leave the pre-invasive stage:

$$N(a) = \int_0^{a-a_0} f(z)R(a\text{-}z)dz \quad (4)$$

Now the detection rate of pre-invasive lesions at a first screening at age $a$ is:

$$P_F(a) = rr\, s_P \int_0^{a} (R(x) - N(x))dx \quad (5)$$

And the detection rate of invasive pre-clinical lesions:

$$P_I(a) = rr\, s_I \int_0^a (N(x) - I(x))dx \qquad (6)$$

The detection rate of pre-clinical lesions for a second smear at age $u_2$ depends on the age $u_1$ at which the first smear was made.

$$S(u_1,u_2) = \int_{a_0}^{u_2} rr\, R(a)\left[s_P\{1\text{-}F(u_2-a)\} + s_I p(a)\{F(u_2-a) - G(u_2-a)\}\right] (1-sP)^{a<u_1}\, da \qquad (7)$$

The notation $(1-s_P)^{a<u_1}$ is used to indicate that the false negative rate at the first screening should be taken into account only in cases where the onset occurred before age $u_1$.

For the detection rates of second and later smears, a further simplifying assumption was made to reduce numerical complexity: the fast negative rate at the subsequent smear(s) is assumed to be $1.0-s_P$ for all screen-detectable lesions. Thus, the expression for the false negative rate is an approximation, neglecting the lower false negative rate $1.0-s_I$ in cases who are in stage preclinical invasive already at age $u_2$. This will result in a slight exaggeration of the detection rate, since lesions that are in stage pre-clinical invasive cervical cancer at preceding smears would have a false negative rate of $u_2$.

The detection rate at a third screen at age $u_3$ depends on the ages $u_1$ and $u_2$ of the preceding smears:

$$\begin{aligned} T(u_1,u_2,u_3) = & \int_{a_0}^{u_1} rr\, R(a)(1-s_P)^2\left[s_P\{1-F(u_3-a)\} + s_I p(a)\{F(u_3-a) - G(u_3-a)\}\right]da \\ & + \int_{u_1}^{u_2} rr\, R(a)(1-s_P)\left[s_P\{1-F(u_3-a)\} + s_I p(a)\{F(u_3-a) - G(u_3-a)\}\right]da \\ & + \int_{u_2}^{u_3} rr\, R(a)\left[s_P\{1-F(u_3\text{-}a)\} + s_I p(a)\{F(u_3-a) - G(u_3-a)\}\right]da \qquad (8) \end{aligned}$$

The simplifications in $R(a)$ and $p(a)$ are:

$$R(a) = \begin{cases} r_1 & a_0<a<a_1 \\ r_2 & a>a_1 \end{cases}$$

$$p(a) = \begin{cases} p_1 & a_0<a<a_1 \\ p_2 & a>a_1 \end{cases}$$

Expressions (2), (5), (7) and (8) can now be simplified considerably. The resulting expressions are used to calculate expected rates for a given set of parameter values. The clinical incidence $I(a)$ and the detection rate of a first smear are calculated for 1-year age-groups and then aggregated to the classes used in the testing procedure. The detection rates $S(u_1,u_2)$ of a second smear are calculated for a matrix of 'ages at mid-point of the interval' and intervals. The same method is used for detection rates $T(u_1,u_2,u_3)$ of third and subsequent smears, assuming that the interval between the first and second smear is 1, 2, 3 or 5 years with probabilities 0.40, 0.30, 0.15 and 0.15, respectively (based on women years in the published tables for the second smear). These rates are also aggregated, after dividing the rates by the lengh of the interval to obtain rates that have women-years as denominator. Expected numbers of cases are obtained by multiplying expected rates and observed denominators (women-years or number screened).

The log-likelihood is based on the assumption that the observed cases are a realisation of a Poisson-distribution with mean = expected number of cases. The likelihood is maximised using a downhill simplex multidimensional optimisation routine (Press *et al.*, 1988). The total number of classes is 45, the number of degrees of freedom for the Pearson Chi-Square test for the goodness of fit of the model is 41 minus the number of free parameters that were varied in deriving the maximum likelihood estimates. The number of degrees of freedom equals the number of categories minus 4, since in each cohort both expected and observed sum of cases detected with the second smear and with subsequent smears is the same for the two subclassifications (by age and by interval since first smear), as can be seen in Table A-I. The deviance, i.e. the likelihood ratio test statistic for comparing a model with the complete model, is also inspected in assessing the goodness of fit.

Comparisons between models are based on the Likelihood-ratio test. Also, 95% confidence regions for one and for two parameters are obtained by inverting the Likelihood-ratio test, i.e., by searching for parameter values for which the log likelihood is $3.84 \div 2$ respectively $5.99 \div 2$ lower than the log-likelihood of the optimal model. One- and two-dimensional confidence regions (Table II, Figure 2) are computed by repeatedly applying the downhill simplex optimisation routine in combination with a root-finding algorithm.

### References

ADAMI, H.O. & GUSTAFSSON, L. (1990). Cervical cancer screening (Reply). *Br. J. Cancer*, **62**, 334.

ALBERT, A. (1981). Estimated cervical cancer disease state incidence and transition rates. *JNCI*, **67**, 571.

ANDERSON, G.H., BOYES, D.A., BENEDET, J.L. & 6 others (1988). Organisation and results of the cervical cytology screening programme in British Columbia, 1955–85. *Br. Med. J.*, **296**, 975.

VAN BALLEGOOIJEN, M., KOOPMANSCHAP, M.A., VAN OORTMARSSEN, G.J., HABBEMA, J.D.F., LUBBE, J.Th.N. & VAN AGT, H.M.E. (1990). Diagnostic and treatment procedures induced by cervical cancer screening. *Eur. J. Cancer*, **26**, 941.

BOYES, D.A., MORRISON, B., KNOX, E.G., DRAPER, G.J. & MILLER, A.B. (1982). A cohort study of cervical cancer screening in British Columbia. *Clin. Invest. Med.*, **5**, 1.

BRINTON, L.A. & FRAUMENI, J.F. Jr (1986). Epidemiology of uterine cervical cancer. *J. Chron. Dis.*, **39**, 1051.

BROOKMEYER, R. & DAY, N.E. (1987). Two-stage models for the analysis of cancer screening data. *Biometrics*, **43**, 657.

COPPLESON, L.W. & BROWN, B. (1975). Observations on a model of the biology of carcinoma of the cervix: a poor fit between observation and theory. *Am. J. Obstet. Gynecol.*, **122**, 127.

DAY, N.E. & WALTER, S.D. (1984). Simplified models of screening for chronic disease: estimation procedures from mass screening programmes. *Biometrics*, **40**, 1.

EDDY, D.M. (1981). Appropriateness of cervical cancer screening. *Gynecologic Oncol.*, **12**, S168.

GUSTAFSSON, L. & ADAMI, H.O. (1989). Natural history of cervical neoplasia: consistent results obtained by an identification technique. *Br. J. Cancer*, **60**, 132.

IARC WORKING GROUP ON EVALUATION OF CERVICAL CANCER SCREENING PROGRAMMES (1986). Screening for squamous cervical cancer: duration of low risk after negative results of cervical cytology and its implication for screening policies. *Br. Med. J.*, **293**, 659.

KINLEN, L.J. & SPRIGGS, A.I. (1978). Women with positive cervical smears but without surgical intervention. *Lancet*, **ii**, 463.

KNOX, E.G. (1973). A simulation system for screening procedures. In *The Future and Present Indicatives*, McLachlan, G. (ed.) p. 19–55. Oxford University Press: London.

KOOPMANSCHAP, M.A., LUBBE, J.Th.N., VAN OORTMARSSEN, G.J., VAN AGT, H.M.E., VAN BALLEGOOIJEN, M. & HABBEMA, J.D.F. (1990*a*). Economic aspects of cervical cancer screening. *Social Sci & Med.*, **10**, 1081.

KOOPMANSCHAP, M.A., VAN OORTMARSSEN, G.J., VAN AGT, H.M.E., VAN BALLEGOOIJEN, M., HABBEMA, J.D.F. & LUBBE, J.Th.N. (1990*b*). Cervical-cancer screening: attendance and cost-effectiveness. *Int. J. Cancer*, **45**, 410.

KOTTMEIER, H.L. (1961). Evolution et traitement des epitheliomas. *Rev. Fr. Gynecol. Obstet.*, **56**, 821.

VAN OORTMARSSEN, G.J. & HABBEMA, J.D.F. (1990). Cervical cancer screening (Letter to the editor). *Br. J. Cancer*, **62**, 333.

PRESS, W.H., FLANNERY, B.P., TEUKOLSKY, S.A. & VETTERLING, W.T. (1988). *Numerical Recipes in C. The Art of Scientific Computing*. Cambridge University Press: Cambridge.

PROROK, P.C. (1986). Mathematical models and natural history in cervical cancer screening. In *Screening for Cancer of the Uterine Cervix*. Hakama, M., Miller, A.B. & Day, N.E. (eds), pp. 185–196. International Agency for Research on Cancer: Lyon.